# Effects of aerobic and inspiratory training on skeletal muscle microRNA‐1 and downstream‐associated pathways in patients with heart failure

**DOI:** 10.1002/jcsm.12495

**Published:** 2019-11-19

**Authors:** Ligia M. Antunes‐Correa, Patricia F. Trevizan, Aline V.N. Bacurau, Larissa Ferreira‐Santos, João L.P. Gomes, Ursula Urias, Patricia A. Oliveira, Maria Janieire N.N. Alves, Dirceu R. de Almeida, Patricia C. Brum, Edilamar M. Oliveira, Ludhmila Hajjar, Roberto Kalil Filho, Carlos Eduardo Negrão

**Affiliations:** ^1^ Heart Institute (InCor) University of São Paulo Medical School São Paulo Brazil; ^2^ School of Physical Education and Sport University of São Paulo São Paulo Brazil; ^3^ Division of Cardiology, Department of Medicine Federal University of São Paulo São Paulo Brazil; ^4^ School of Physical Education University of Campinas (UNICAMP) Campinas Brazil

**Keywords:** Aerobic exercise training, Heart failure, Inspiratory muscle training, MicroRNA‐1, Skeletal myopathy

## Abstract

**Background:**

The exercise intolerance in chronic heart failure with reduced ejection fraction (HFrEF) is mostly attributed to alterations in skeletal muscle. However, the mechanisms underlying the skeletal myopathy in patients with HFrEF are not completely understood. We hypothesized that (i) aerobic exercise training (AET) and inspiratory muscle training (IMT) would change skeletal muscle microRNA‐1 expression and downstream‐associated pathways in patients with HFrEF and (ii) AET and IMT would increase leg blood flow (LBF), functional capacity, and quality of life in these patients.

**Methods:**

Patients age 35 to 70 years, left ventricular ejection fraction (LVEF) ≤40%, New York Heart Association functional classes II–III, were randomized into control, IMT, and AET groups. Skeletal muscle changes were examined by vastus lateralis biopsy. LBF was measured by venous occlusion plethysmography, functional capacity by cardiopulmonary exercise test, and quality of life by Minnesota Living with Heart Failure Questionnaire. All patients were evaluated at baseline and after 4 months.

**Results:**

Thirty‐three patients finished the study protocol: control (*n* = 10; LVEF = 25 ± 1%; six males), IMT (*n* = 11; LVEF = 31 ± 2%; three males), and AET (*n* = 12; LVEF = 26 ± 2%; seven males). AET, but not IMT, increased the expression of microRNA‐1 (*P* = 0.02; percent changes = 53 ± 17%), decreased the expression of PTEN (*P* = 0.003; percent changes = −15 ± 0.03%), and tended to increase the p‐AKT^ser473^/AKT ratio (*P* = 0.06). In addition, AET decreased HDAC4 expression (*P* = 0.03; percent changes = −40 ± 19%) and upregulated follistatin (*P* = 0.01; percent changes = 174 ± 58%), MEF2C (*P* = 0.05; percent changes = 34 ± 15%), and MyoD expression (*P* = 0.05; percent changes = 47 ± 18%). AET also increased muscle cross‐sectional area (*P* = 0.01). AET and IMT increased LBF, functional capacity, and quality of life. Further analyses showed a significant correlation between percent changes in microRNA‐1 and percent changes in follistatin mRNA (*P* = 0.001, rho = 0.58) and between percent changes in follistatin mRNA and percent changes in peak VO_2_ (*P* = 0.004, rho = 0.51).

**Conclusions:**

AET upregulates microRNA‐1 levels and decreases the protein expression of PTEN, which reduces the inhibitory action on the PI3K‐AKT pathway that regulates the skeletal muscle tropism. The increased levels of microRNA‐1 also decreased HDAC4 and increased MEF2c, MyoD, and follistatin expression, improving skeletal muscle regeneration. These changes associated with the increase in muscle cross‐sectional area and LBF contribute to the attenuation in skeletal myopathy, and the improvement in functional capacity and quality of life in patients with HFrEF. IMT caused no changes in microRNA‐1 and in the downstream‐associated pathway. The increased functional capacity provoked by IMT seems to be associated with amelioration in the respiratory function instead of changes in skeletal muscle.

http://ClinicalTrials.gov (Identifier: NCT01747395)

## Introduction

Chronic heart failure with reduced ejection fraction (HFrEF) is the major cause of morbidity and mortality in the developed countries.^1^ In the last decades, this scenario has also reached the developing countries, placing the treatment of this syndrome among the most expensive healthcare costs worldwide.^1^

Chronic heart failure is characterized by compensatory neurohumoral activation: renin angiotensin aldosterone system and sympathetic nervous system are substantially activated in HFrEF. These alterations provoke vasoconstriction and muscle underperfusion, which chronically contribute to skeletal muscle metabolic disturbances, including increase in reactive oxygen species, inflammation, shift toward fast‐twitch fibres, and imbalance in skeletal muscle protein degradation and synthesis. All these changes contribute to skeletal myopathy, which results in reduced muscle contraction force and exercise tolerance in patients with HFrEF.^2^, ^3^, ^4^, ^5^ The investigation of the skeletal myocyte micro ambient and its mechanism of control is crucial to understand the skeletal myopathy in chronic heart failure and, of course, to provide knowledge for the treatment of patients suffering from this syndrome.

MicroRNAs are genetically conserved small non‐coding RNAs, containing about 22 nucleotides that post‐transcriptionally control gene expression. Skeletal muscle‐enriched microRNAs (myomiRs) play an important role in the regulation of muscle development, growth, regeneration, and metabolism.^6^, ^7^, ^8^, ^9^ The microRNA‐1 and microRNA‐133a can regulate myogenesis by suppressing the predicted target gene *histone deacetylase 4* (HDAC4), which upregulates myogenic markers as *myocyte enhancer factor 2C* (MEF2c), *myogenic differentiation factor D* (MyoD), and *follistatin* expression.^10^, ^11^, ^12^ Whether alterations in microRNA‐1 and microRNA‐133a and downstream‐associated pathways contribute to skeletal myopathy in patients with chronic HFrEF is unknown.

Exercise‐based cardiac rehabilitation has been strongly recommended in the management of HF patients.^13^ This intervention improves functional capacity, exercise intolerance, and quality of life in patients with HFrEF.^14^ However, it remains unclear whether exercise training impacts the risk of mortality and hospitalization in this set of patients.^15^ The clinical improvement in exercise‐trained patients with HFrEF has been associated with changes in neurovascular control, inflammatory response, and amelioration in skeletal myopathy.^5^ Aerobic exercise training (AET) has been shown to provoke a remarkable reduction in muscle sympathetic nerve activity and vasoconstriction.^16^, ^17^, ^18^, ^19^, ^20^, ^21^, ^22^, ^23^, ^24^, ^25^ In skeletal muscle, AET reduces inflammation, oxidative stress, and energy metabolism and improves the balance between muscle protein synthesis and degradation.^3^, ^5^, ^26^, ^27^ Moreover, there are data supporting the notion that AET can increase and/or decrease skeletal muscle myomiRs and, in consequence, alter skeletal muscle phenotype in cardiovascular diseases.^28^, ^29^, ^30^, ^31^, ^32^

Previous studies show that inspiratory muscle training (IMT) decreases sympathetic nerve activity and improves muscle blood flow and functional capacity in patients with HFrEF.^33^, ^34^, ^35^, ^36^, ^37^, ^38^, ^39^ However, the effects of IMT on skeletal muscle myopathy in patients with HFrEF remain unknown.

In this study, we hypothesized that (i) AET and IMT will change the expression of skeletal muscle microRNA‐1 and microRNA‐133a, and the downstream‐associated pathways in patients with chronic HFrEF and (ii) AET and IMT will increase leg blood flow, functional capacity, and quality of life in these patients.

## Methods

### Study population

Patients diagnosed with HF, age 35 to 70 years, reduced left ventricular ejection fraction (≤40%), New York Heart Association functional classes II–III, peak oxygen uptake ≤20 mL/kg/min, body mass index ≤35 kg/m^2^ treated with guideline‐directed medical therapy for HFrEF were invited to participate in the study. The exclusion criteria were patients with severe pulmonary, neurologic, or orthopaedic disease, neoplasia, end‐stage renal failure on dialysis, insulin‐dependent diabetes mellitus, acute myocardium infarction or heart surgery in the last 6 months, unstable angina, atrial fibrillation, current tobacco smoker, pregnancy, and participation on a formal exercise training programme. Patients that were hospitalized, or died, during the study protocol were excluded from the final analysis. The patients were randomized into three groups: (i) control, (ii) IMT, and (iii) AET. All patients were evaluated at baseline and after 4 months. The minimum required attendance in the exercise training sessions for inclusion in the study was 75%. The study was conducted in accordance with the Declaration of Helsinki. The study was approved by the Research Committee of the Heart Institute (SDC#3565/10/154) and Human Subject Protection Committee at the Clinical Hospital of the School of Medicine of the University of São Paulo (CAPPesq:814‐10). All subjects provided written informed consent prior to participation in the study. This trial is registered at http://ClinicalTrials.gov (*Identifier*: *NCT01747395*).

### Sample size and power calculations

The sample size was calculated based upon a previous study,^18^ which demonstrated that to detect an 18% increase in peak VO_2_ at least 11 patients would be needed for each group. We included 30% more patients in each intervention groups because of dropouts observed in our previous studies. The alpha error was defined as 0.05 with the power of 80%.

### Measures and procedures

#### Leg blood flow

Leg blood flow (LBF) was measured by venous occlusion plethysmography as previously described.^40^ Briefly, the left leg was elevated above heart level to ensure adequate venous drainage. A mercury‐filled silastic tube attached to a low‐pressure transducer was placed around the calf and connected to plethysmography (Hokanson‐AI‐6, USA). Sphygmomanometer cuffs were placed around the thigh and ankle. At 20 s intervals, the thigh cuff was inflated above venous pressure for 10 s. Leg vascular conductance (LVC) was calculated by dividing LBF by mean arterial pressure, times 100.

#### Respiratory muscle strength

Respiratory muscle strength was determined by maximal inspiratory pressure was assessed by means of a pressure transducer (MVD500‐Globalmed, Brazil) as previously described.^41^

#### Functional capacity

Maximal exercise capacity was determined during a cardiopulmonary exercise testing on a cycle ergometer (Ergoline‐Spirit150, DEU), using a ramp protocol with work rate increments of 5–10 W every minute until exhaustion as previously described.^16^, ^22^, ^25^ Briefly, metabolic parameters, as oxygen uptake (VO_2_) and carbon dioxide production, were determined by means of gas exchange on a breath‐by‐breath basis in a computerized system (Sensor Medics, Model‐Vmax‐229, USA). Peak VO_2_ was defined as the maximum attained VO_2_ at the end of the exercise period when the patient could no longer maintain the cycle ergometer velocity at 60 rpm. Anaerobic threshold was determined to occur at the breakpoint between the increase in the carbon dioxide output and VO_2_ (V‐slope) or at the point in which the ventilatory equivalent for oxygen and end‐tidal oxygen partial pressure curves reached their respective minimum values and began to rise. Respiratory compensation was determined to occur at the point at which ventilatory equivalent for carbon dioxide was lowest before a systematic increase and when end‐tidal carbon dioxide partial pressure reaches a maximum value and begins to decrease.^42^ In addition, we evaluated the peak workload (Watts) at end of the exercise.

#### Quality of life

Quality of life was evaluated by means of Minnesota Living with Heart Failure Questionnaire (MLHFQ).^43^

#### Other measurements

Left ventricular ejection fraction was determined from the two‐dimensional echocardiography by Simpson method (IE33‐Philips Medical Systems, USA). Heart rate, systolic, diastolic, and mean blood pressure were noninvasively evaluated on a beat‐to‐beat basis by means of a finger photoplethysmography (Finometer‐Pro, Finapres Medical Systems, NED).^18^

#### Skeletal muscle biopsy

Percutaneous muscle biopsy procedures were performed in vastus lateralis, approximately at the midway point between the top edge of the patella and the greater trochanter. The volunteers assumed a comfortable reclining position with both legs out stretched. After local asepsis with chlorhexidine 0.5% (alcoholic solution), skin and subcutaneous tissue were infiltrated with 5–10 mL of 1% lidocaine. After ensuring adequate local anaesthesia, a small incision was made in the skin and subcutaneous tissue (0.5 cm in length and 1 cm in depth). The local bleeding was stanched by compression. A 5‐mm modified Allendale‐Bergstrom needle was then inserted through the fascia, and an assistant immediately applied suction by using a syringe connected to a canister and attached to the top of the needle. A muscle sample was removed, and the skin was closed with skin closure tape (*Steri‐Strip – 3M*). A pressure dressing was immediately applied and maintained for 24 h.^18^, ^44^ The muscle sample was immediately frozen in liquid nitrogen and subsequently stored in a freezer at −80°C.

#### MicroRNA and mRNA analysis by real‐time polymerase chain reaction

MicroRNA and mRNA levels in the vastus lateralis muscle were analysed by real‐time polymerase chain reaction method, as previously described.^18^ Frozen skeletal muscle samples were homogenized in Trizol, and RNA was isolated according to the manufacturer's instructions (Invitrogen Life Technologies, USA). After extraction, the total RNA concentration was quantified using NanoDrop Spectrophotometer (NanoDrop Technologies, USA) and checked for integrity by EtBr‐agarose gel electrophoresis. RNA was primed with 0.5 μg/μL oligo dT (Fermentas/Thermo Scientific Molecular Biology, USA) to generate first strand DNA. Reverse transcription was performed using Revertaid M‐MuLV Reverse Transcriptase (Fermentas/Thermo Scientific Molecular Biology, USA). cDNA for microRNA analysis was synthesized from total RNA using gene‐specific primers according to the TaqMan MicroRNA Assay protocol (Applied Biosystems, USA). MicroRNA levels of miRNA‐1 (Life Technologies, #INV002222) and miRNA‐133a (Life Technologies, #INV002246) were performed using TaqMan MicroRNA Assay protocol (Applied Biosystems, USA) and normalized by evaluating U6 expression. mRNA levels of the follistatin (#FW:5‐CTGCTGCTCTGCCAGTTCAT‐3; RV:5‐CCTTGCTCAGTTCGGTCTTGTA‐3#), MEF2c (#FW:5‐TTCTCCTCCTAGAGACCGTACCA‐3; RV:5‐CGTGGCGCGTGTGTTGT‐3#), and MyoD (#FW:5‐CCGACGGCATGATGGACTA‐3; RV 5‐TGGGCGCCTCGTTGTAGTA‐3#) were performed with a SYBRGreen PCR Master Mix protocol and normalized by 18S (#FW:5‐GTAACCCGTTGAACCCCATT‐3; RV 5‐CCATCCAATCGGTAGTAGCG‐3#) as an internal control. Primers were designed using Primer‐BLAST. Real‐time quantification was performed using ABI PRISM 7700 Sequence Detection System (Applied Biosystem, USA). Results were quantified as CT values, where CT is defined as the threshold cycle of the polymerase chain reaction at which the amplified product is first detected. Relative quantities of microRNA and target gene were normalized by the values of the reference gene (ΔCT). Fold changes in microRNA and mRNA expression were calculated using the differences in ΔCT values between the two samples (ΔΔCT) and equation 2^−ΔΔCT^. Results are expressed as % of pre for each sample.

#### Expression of protein levels analysis by western blot

The protein levels of phosphatase and tensin homolog (PTEN), phosphoinositide 3‐kinase (PI3K), protein kinase B (AKT), phospho‐AKT (p‐AKT), and phospho‐HDAC4 (p‐HDAC4) in the vastus lateralis muscle biopsy were analysed by western blotting, as previously described.^45^, ^46^ Frozen samples were homogenized in cell lysis buffer (100 mM Tris‐HCl, 50 mM NaCl, 1% Triton X‐100) and protease and phosphatase inhibitor cocktail (1:100; Sigma‐Aldrich, USA). Vastus lateralis tissue debris was removed by centrifugation at 3000 × *g*, 4°C, 10 min. Samples were loaded and subjected to SDS‐PAGE on polyacrylamide gels (8–10%) depending on the protein molecular weight. After electrophoresis, proteins were electrotransferred to a nitrocellulose membrane (BioRad Biosciences, USA). Equal loading of samples (30 μg) and even transfer efficiency were monitored with the use of 0.5% Ponceau staining of the blot membrane. The blot membrane was then incubated in a blocking buffer (5% nonfat dry milk, 10 mM Tris‐HCl (pH 7.6), 150 mM NaCl, and 0.1% Tween 20) for 2 h at room temperature and then incubated overnight at 4°C with antiPten (Cell Signalling, #9552), anti‐PI3K (Cell Signalling, #4255), anti‐AKT (Cell Signalling, #2938), anti‐p‐AKT^Ser473^ (Cell Signalling, #9271), and anti‐p‐HDAC4^Ser632^ (Abcam, ab39408) polyclonal antibodies. Binding of the primary antiPTEN was detected by peroxidase conjugated secondary antibodies (Invitrogen, #65‐6120), and enhanced chemiluminescence reagent (Amersham Biosciences, USA) and detection were performed in a digitalizing unit by ChemiDoc (BioRad, USA). The bands were analysed using ImageJ software (ImageJ Corporation based on NIH image). Binding of the primary anti‐PI3K, anti‐AKT, anti‐p‐AKT, and anti‐HDAC4 was detected by secondary antibody (IRDye 800CW anti‐rabbit, LI‐COR Biosciences, #926‐32211), and detection was performed by LI‐COR (LI‐COR Biosciences, USA). The bands were analysed using Image Studio Lite software (LI‐COR Biosciences, USA). The results of protein expression were normalized by Ponceau staining. They are expressed as a percentage of pre expression for each sample.

#### Fibre cross‐sectional area analysis by immunohistochemistry

The muscle samples collected at the time of biopsy were embedded in Tissue‐Tek® (Sakura, USA), frozen in isopentane and then in liquid nitrogen. Muscle fragments were sectioned in a cryostat (10 μm thick; Leica‐CM1850, Leica Microsystems, DEU). Fixed muscle sections were submitted to immunohistochemistry for laminin (Abcam, ab7784) to analyse the cross‐sectional area. The muscle sections were fixed with 4% formalin (Sigma‐Aldrich, HT501128) for 10 min at room temperature, permeabilized in 0.2% Triton X‐100 (Bio‐Rad, 01‐0407) and 1% bovine serum albumin (Amresco, E588) diluted in phosphate buffer saline (PBS; Sigma‐Aldrich, P4417) for 10 min. Blocking was performed with 10% goat serum (Sigma‐Aldrich, G9023) in PBS for 45 min. Glass slides were incubated with a solution containing the primary antibody to laminin (dilution 1:100) for delimited muscle fibres, with 1.5% goat serum in PBS for 1 h 30 min at room temperature. After proper washing, the sections were incubated with respective fluorescent secondary antibodies to laminin (#A‐11008, Invitrogen). The images were captured on a computer attached to a fluorescent microscope and connected to a photographic system (magnification, 200×) (Leica QWin, Leica Microsystems, DEU). Skeletal muscle cross‐sectional area of each fibre was evaluated by Image J software (Image J Corporation based on NIH image). The results are expressed as the mean of the cross‐sectional area of all fibres captured for each sample.

#### Inspiratory muscle training

Inspiratory muscle training (IMT) was conducted for 30 min, five times a week, for 4 months using a resistive loading device (POWERbreathe‐Plus®, POWERbreathe International Limited, UK). All patients exercised at 60% percentage of individual MPI, measured once a week. Patients were instructed to maintain diaphragmatic breathing at a rate of 15 to 20 breaths/min. Four training sessions per week were performed at home and one training session at Heart Institute under supervision.^35^

#### Aerobic exercise training

Moderate AET was conducted for 4 months at the Heart Institute, School of Medicine, University of São Paulo, under supervision. It consisted of three sessions per week. Each session included 5 min stretching exercises, 40 min of cycling, 10 min of local strengthening exercises, and 5 min of cool down. The aerobic exercise was conducted at anaerobic threshold up to 10% below the respiratory compensation point. The bicycle workload was increased in 0.25 or 0.5 kpm when exercise training adaptation occurred. Aerobic exercise duration increased progressively so that all patients could perform 40 min of cycling at the established intensity.^18^

### Statistical analysis

The data are presented as means ± SE. The Kolmogorov–Smirnov and Levene's test was used to assess the normality of distribution and homogeneity for each variable. Parametric tests were used for variables with normal distribution and homogeneity. The effects of AET and IMT were verified by Student's *t*‐test for paired data in each group. In addition, the delta changes between 4 months at baseline were verified by one‐way analysis of variance followed by Scheffé's post hoc multiple comparisons. Nonparametric tests were used for variables with no normal distribution and homogeneity (Kruskal–Wallis test and Mann–Whitney's test). A χ^2^ test was used to assess categorical data differences. Spearman correlation analysis was performed to test correlation between molecular percent changes and peak VO_2_ percent changes. Probability values of *P* ≤ 0.05 were considered statistically significant

## Results

### Study population

**Figure 1:**
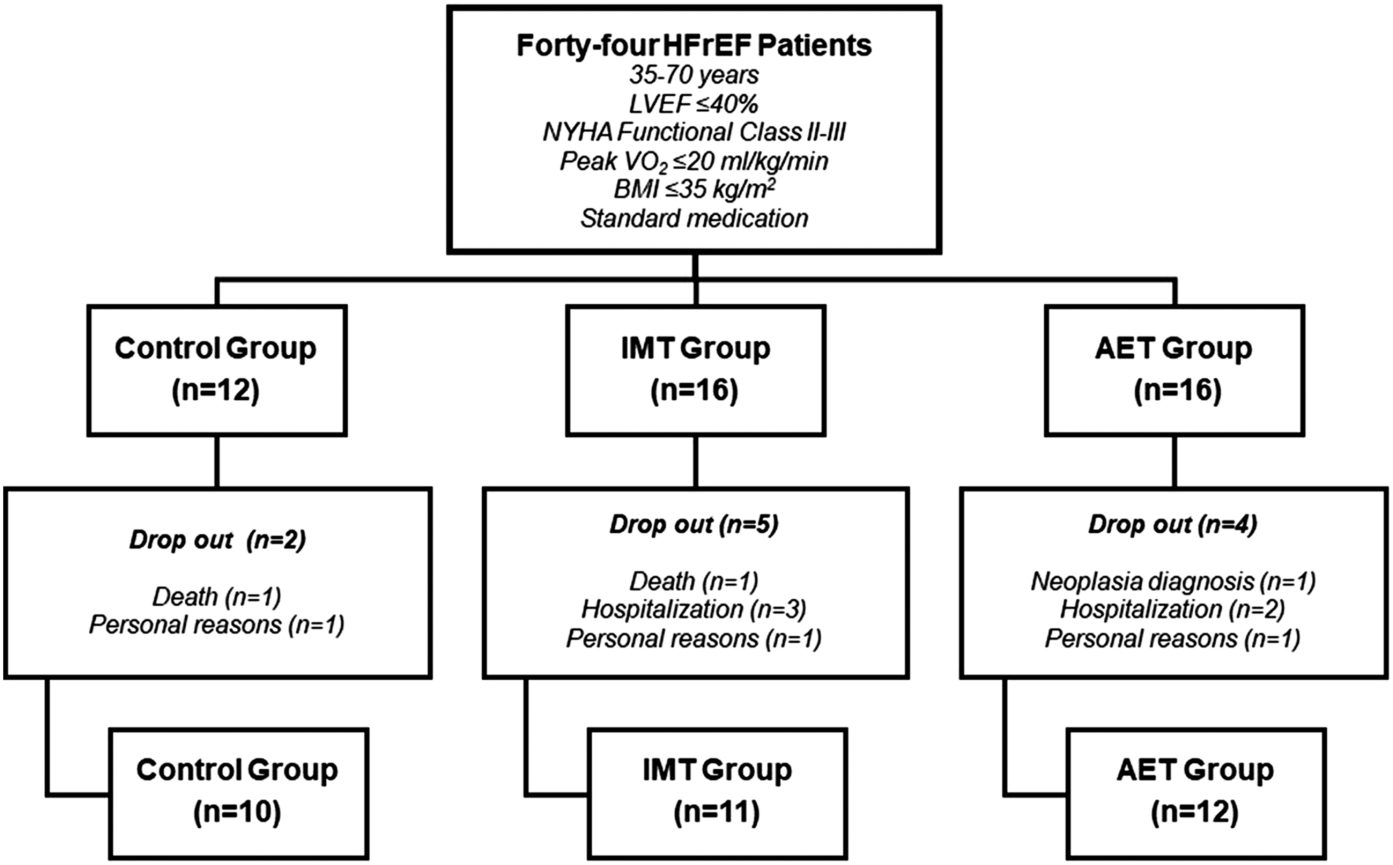
Study design. AET, aerobic exercise training; BMI, body mass index; HFrEF, heart failure with reduced ejection fraction; IMT, inspiratory muscle training; LVEF, left ventricular ejection fraction; NYHA, New York Heart Association; VO_2_, oxygen uptake.

After screening, 44 HFrEF patients were randomized into three groups: (i) control group (*n* = 12), (ii) IMT group (*n* = 16), and (iii) AET group (*n* = 16). In the control group, two patients did not complete the study because of death (*n* = 1) and drop out for personal reasons (*n* = 1). In the IMT group, five patients did not complete the training protocol because of death (*n* = 1), hospitalization [HF decompensation (*n* = 1); other cardiovascular events (*n* = 2)], and drop out for personal reasons (*n* = 1). In the AET group, four patients did not complete the training protocol because of hospitalization [HF decompensation (*n* = 1); other cardiovascular events (*n* = 1)], cancer diagnosis (*n* = 1), and drop out for personal reasons (*n* = 1). Thus, data are available for analysis in 33 subjects (*Figure* 1).

### Baseline measurements

Baseline characteristics of heart failure patients who completed the study are shown in *Table* 1. There were no differences among groups in physical characteristics, gender, functional class, aetiology, medications, associated comorbidities, haemodynamic parameters, respiratory muscle strength, functional capacity, and quality of life.

**Table 1:** Baseline characteristics in patients with heart failure selected to inspiratory muscle training, aerobic exercise training, and control groups

	Control (*n* = 10)	IMT (*n* = 11)	AET (*n* = 12)	*P*
Physical characteristics				
Age (year)	57 ± 3	55 ± 3	57 ± 2	0.83
Weight (kg)	72 ± 4	77 ± 6	76 ± 6	0.79
BMI (kg/m^2^)	27 ± 1	29 ± 2	28 ± 1	0.63
Gender				
Male (*n*)	6	3	7	0.23
Female (*n*)	4	8	5	
Functional class				
NYHA‐II (*n*)	9	8	9	0.58
NYHA‐III (*n*)	1	3	3	
HF aetiology				
Ischaemic (*n*)	6	1	4	0.25
Hypertensive (*n*)	2	4	2	
Idiopathic (*n*)	1	5	3	
Chagasic (*n*)	1	0	2	
Others (*n*)	0	1	1	
Medications				
Beta‐blocker (n)	10	11	9	1.00
ACEI/ARB (*n*)	10	11	9	1.00
Spironolactone (*n*)	10	10	12	0.36
Diuretics (*n*)	9	11	12	0.31
Statins (*n*)	6	6	5	0.67
ASA (*n*)	6	3	5	0.32
Digoxin (*n*)	1	4	3	0.37
Hypoglycaemic drugs (*n*)	3	2	2	0.72
Comorbidities				
Diabetes (*n*)	3	2	2	0.72
Dyslipidaemia (*n*)	6	6	6	0.90
Hypertension (*n*)	8	7	9	0.69
Haemodynamic parameters				
LVEF (%)	25 ± 1	31 ± 2	26 ± 2	0.10
HR (beats/min)	68 ± 3	61 ± 2	65 ± 2	0.22
SBP (mmHg)	121 ± 4	121 ± 5	121 ± 6	0.99
DBP (mmHg)	66 ± 2	68 ± 4	69 ± 3	0.72
MBP (mmHg)	86 ± 3	87 ± 4	88 ± 5	0.92
LBF (mL/min/100 mL_tec)	1.86 ± 0.26	1.84 ± 0.20	1.51 ± 0.20	0.45
LVC (u.a.)	2.14 ± 0.31	2.03 ± 0.20	1.69 ± 0.22	0.39
Respiratory muscle function and functional capacity				
Max IP (cmH_2_O)	85 ± 8	86 ± 9	87 ± 10	0.99
Peak VO_2_ (mL/kg/min)	16 ± 1	16 ± 1	15 ± 1	0.80
AT VO_2_ (mL/kg/min)	12 ± 1	11 ± 1	10 ± 1	0.32
Workload peak (Watts)	72 ± 6	75 ± 8	76 ± 10	0.93
O_2_ pulse (mL)	9 ± 1	11 ± 1	10 ± 1	0.51
Total time (seg)	523 ± 50	576 ± 54	542 ± 49	0.78
Quality of life				
MLHFQ (u.a.)	55 ± 6	50 ± 6	56 ± 2	0.64

Values are mean ± SE.

ACEI, angiotensin‐converting enzyme inhibitors; AET, aerobic exercise training; ARB, angiotensin II receptor blocker; ASA, acetylsalicylic acid; AT, anaerobic threshold; BMI, body mass index; DPB, diastolic blood pressure; HR, heart rate; IMT, inspiratory muscle training; LBF, leg blood flow; LVC, leg vascular conductance; LVEF, left ventricular ejection fraction; MBP, mean blood pressure; Max IP, maximal inspiratory pressure; MLHFQ, Minnesota Living with Heart Failure Questionnaire; NYHA, New York Heart Association; SBP, systolic blood pressure; VO_2_, oxygen uptake.

### Effects of aerobic exercise training and inspiratory muscle training on skeletal myopathy

Skeletal muscle biopsy with good quality, pre and post interventions, or clinical care was attained in 29 patients (control, *n* = 10; IMT, *n* = 10; and AET, *n* = 9). The protein expression evaluation and genic expression evaluation were performed in all 29 patients. Muscle tissue to evaluate fibre cross‐sectional area was available in four patients from each group.

#### MicroRNA‐1 and microRNA‐133a

**Figure 2:**
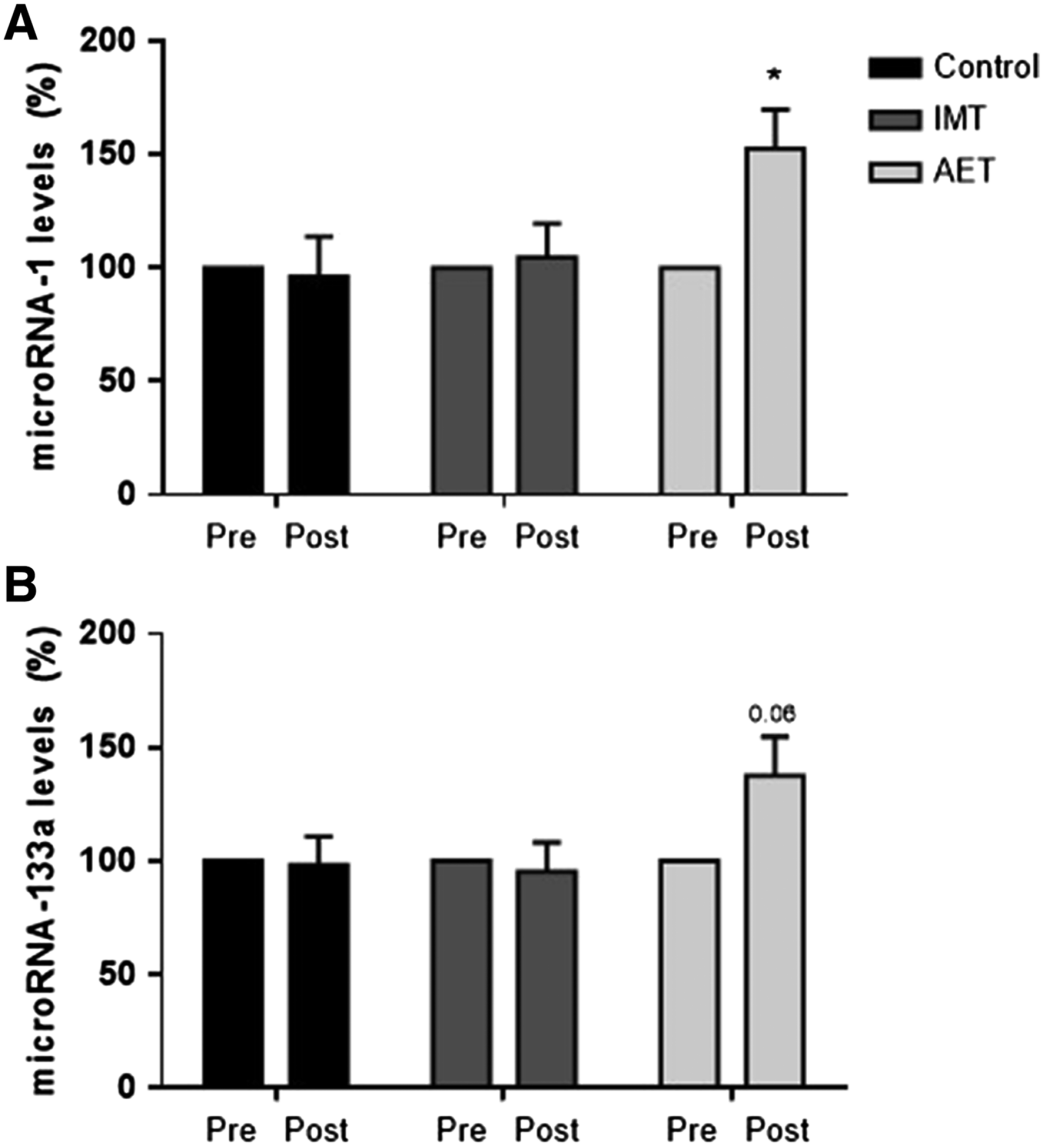
Expression of (A) microRNA‐1 and (B) microRNA‐133a expressed in percent changes (post vs. pre) in control group (control, *n* = 10), inspiratory muscle training group (IMT, *n* = 10), and aerobic exercise training group (AET, *n* = 9). Note that AET provokes a significant increase in expression of microRNA‐1. Values are means ± SE. * vs. pre; *P* < 0.05.

Aerobic exercise training (AET) significantly increased microRNA‐1 levels (*P* = 0.02, *Figure* 2A) and tended to increase microRNA‐133a levels (*P* = 0.06, *Figure* 2B). No changes were found in the control group and the IMT group. The percent change comparisons (post–pre) among groups showed that microRNA‐1 levels tended to be greater in the AET group when compared with control group (*P* = 0.07, *Figure* 2A). No significant differences in microRNA‐133a among groups were found. Despite the structural similarity in these microRNAs, they have different actions in myogenesis. MicroRNA‐1 modulates myoblast differentiation and regeneration, while microRNA‐133a modulates myoblast proliferation, fusion, regeneration, and, muscle fibre shift. Because the microRNA‐1 levels, but not the microRNA‐133a levels, were significantly changed by AET, further investigations were focused on microRNA‐1 downstream‐associated pathways.

#### MicroRNA‐1 downstream pathway: PTEN, PI3K, and AKT

**Figure 3:**
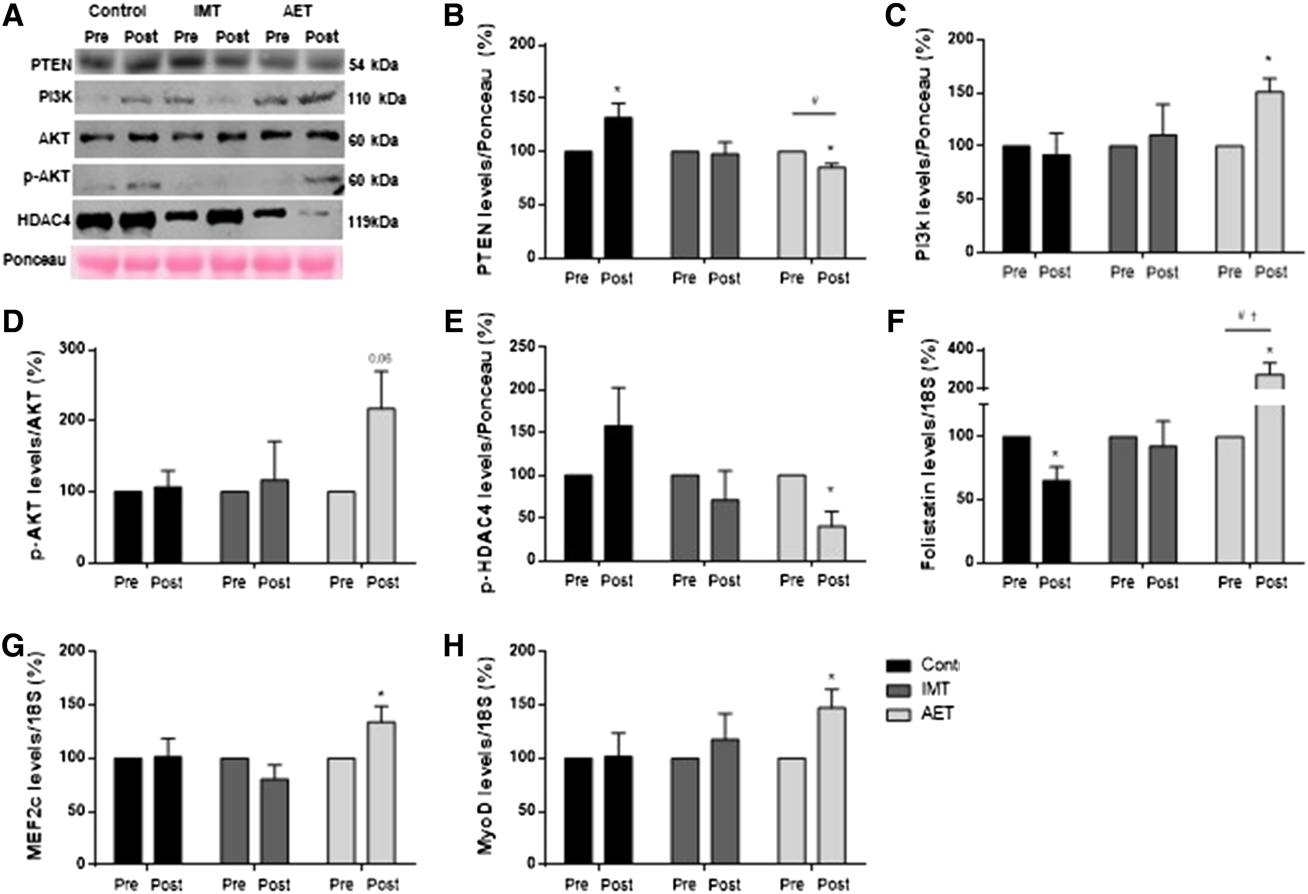
(A) Representative western blots for statistical results, protein expression of (B) PTEN, (C) PI3K, (D) ratio of phospho‐AKT^(ser473)^/AKT, and (E) p‐HDAC4^ser632^. Gene expression of (F) follistatin, (G) MEF2c, and (H) MyoD. The results of protein expression were normalized by Ponceau staining. They are expressed as a percentage of pre expression for each sample in control group (control, *n* = 10), inspiratory muscle training group (IMT, *n* = 10), and aerobic exercise training group (AET, *n* = 9). Note that AET reduces PTEN protein levels, increases PI3K protein levels, and tends to increase p‐AKT^ser473^/AKT ratio (*P* = 0.06). In addition, AET reduces the protein expression of p‐HDAC4^ser632^, increases follistatin mRNA levels, increases MEF2c, and increases MyoD mRNA levels. Values are means ± SE. * vs. pre, ^#^ vs. control group (percent changes), and ^†^vs. IMT group (percent changes); *P* < 0.05.

Aerobic exercise training (AET) significantly reduced PTEN protein levels (*P* = 0.003, *Figure* 3B). In contrast, PTEN protein levels increased in the control group (*P* = 0.05, *Figure* 3B). No changes were found in the IMT. The percent change comparisons among groups showed that the changes caused by AET in the PTEN protein levels were significantly different from those observed in the control group (*P* = 0.02, *Figure* 3B). AET significantly increased PI3K protein levels (*P* = 0.01, *Figure* 3C). No changes in PI3K protein levels in the control and IMT groups were found. AET tended to increase p‐AKT^ser473^/AKT ratio (*P* = 0.06, *Figure* 3D). No changes in p‐AKT^ser473^/AKT ratio in the control and IMT groups were found.

#### MicroRNA‐1 downstream pathway: HDAC4, follistatin, MEF2c, and MyoD

To further explore the effects of AET and IMT on microRNA‐1 downstream pathway, we assessed p‐HDAC4^ser632^, follistatin, MEF2c, and MyoD. AET significantly reduced the protein expression of p‐HDAC4^ser632^ (*P* = 0.03, *Figure* 3E). No significant changes in protein expression of p‐HDAC4^ser632^ were found in the IMT and control groups. Further analysis showed that the percent changes in p‐HDAC4^ser632^ protein levels tended to be different in the AET group compared with the control group (*P* = 0.08, *Figure* 3E). Follistatin mRNA levels were significantly reduced in the control group (*P* = 0.01, *Figure* 3F). In contrast, follistatin mRNA levels were significantly increased in the AET (*P* = 0.02, *Figure* 3F). No changes were found in the IMT group. Further analysis showed that the percent changes in follistatin mRNA levels were significantly greater in the AET group than those observed in the control group and IMT group (*P* = 0.001 and *P* = 0.004, respectively, *Figure* 3F). AET significantly increased MEF2c (*P* = 0.05, *Figure* 3G) and MyoD (*P* = 0.05, *Figure* 3H) mRNA levels. No changes in MEF2c and MyoD mRNA levels were found in the control and IMT groups. Further analyses showed a significant correlation between percent changes in microRNA‐1 and percent changes in follistatin mRNA (*P* = 0.001, rho = 0.58).

#### Fibre cross‐sectional area

**Figure 4:**
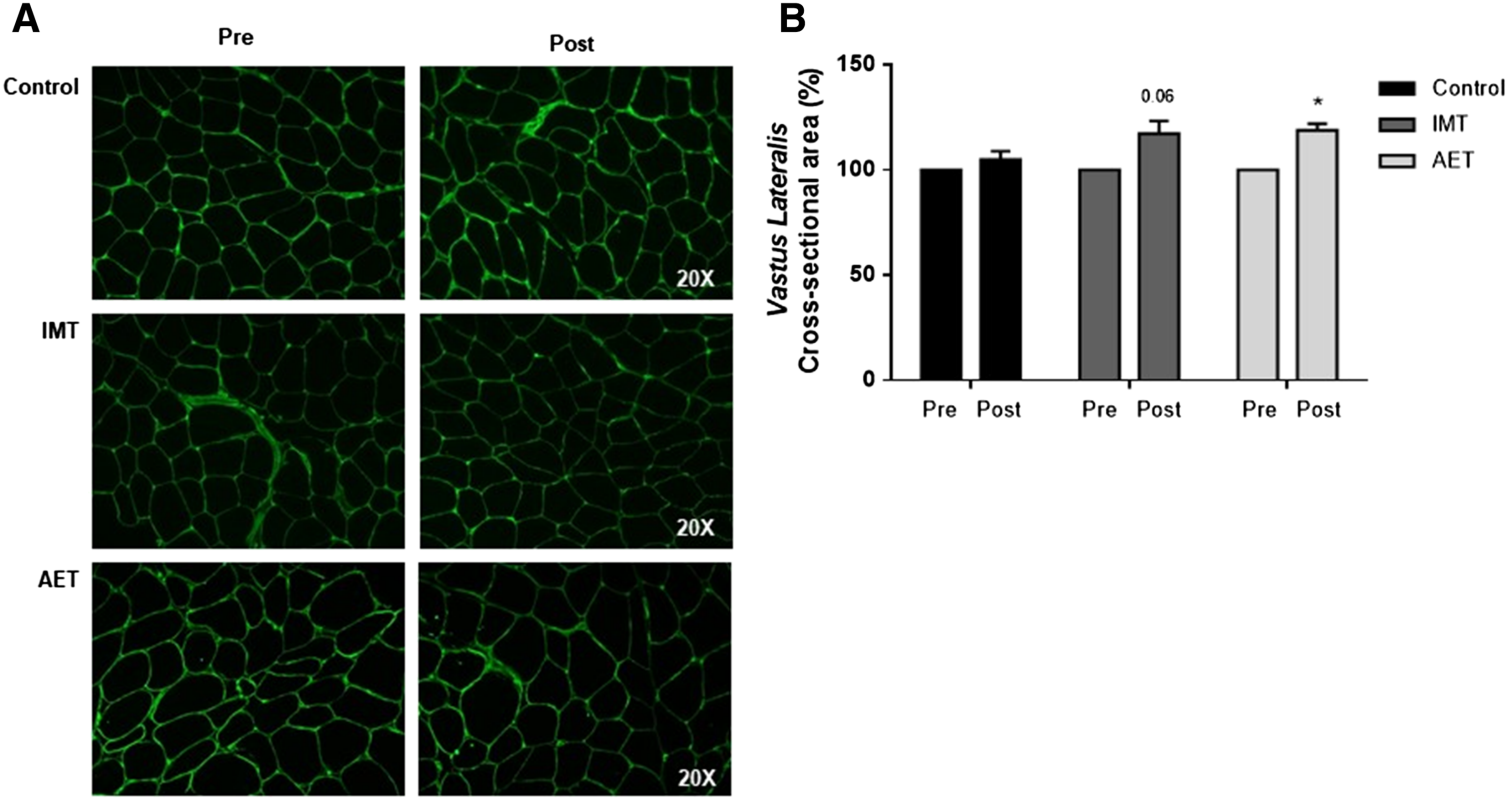
(A) Example of cross‐sectional area of vastus lateralis muscle by one patient from control group (control), inspiratory muscle training group (IMT), and aerobic exercise training group (AET). (B) Cross‐sectional area of vastus lateralis expressed in percent changes (post vs. pre) in control (*n* = 4), IMT (*n* = 4), and AET (*n* = 4). Note that AET increases muscle fibre cross‐sectional area. Values are means ± SE. * vs. pre and ^#^ vs control group (percent changes); *P* < 0.05.

The results regarding the muscle cross‐sectional area are shown in *Figure* 4. AET significantly increased muscle fibre cross‐sectional area (*P* = 0.01). IMT increased muscle fibre cross‐sectional area, but these changes did not achieve significant levels (*P* = 0.06). No changes were found in the control group.

### Effects of aerobic exercise training and inspiratory muscle training on leg blood flow, respiratory muscle strength, physical capacity, and quality of life

**Figure 5:**
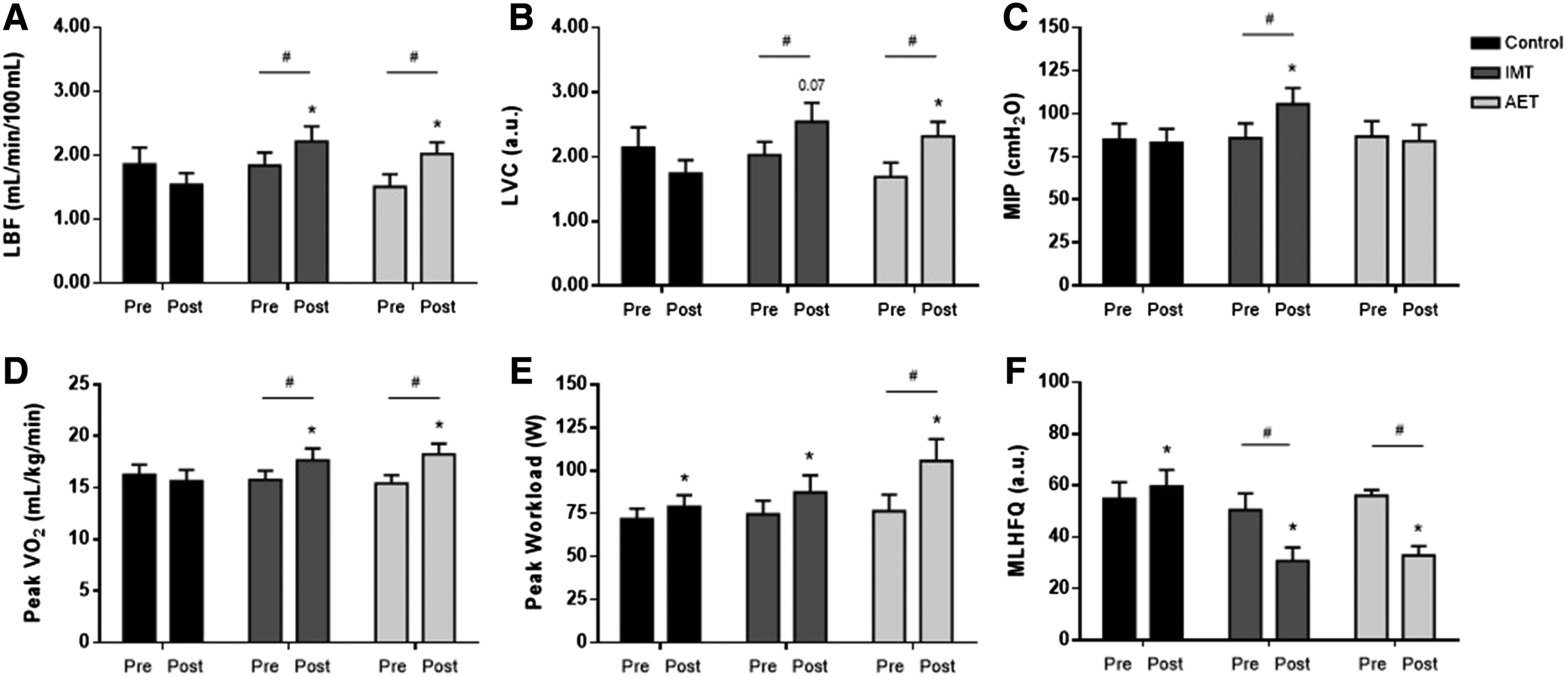
(A) Leg blood flow (LBF), (B) leg vascular conductance (LVC), (C) maximal inspiratory pressure, (D) peak oxygen consumption (peak VO_2_), (E) peak workload, and (F) Minnesota Living with Heart Failure Questionnaire (MLHFQ) score in control group (control, *n* = 10), inspiratory muscle training group (IMT, *n* = 11), and aerobic exercise training group (AET, n = 12). Note that AET and IMT increase LBF and LVC and that the magnitude of changes in LVC is more pronounced in AET group. IMT increases maximal inspiratory pressure. Both AET and IMT increase peak VO_2_. The peak workload increases in the three groups studied. However, the changes in peak workload in AET group were greater than those found in IMT and control groups. Finally, AET and IMT decrease MLHFQ score. Values are means ± SE. * vs. pre and ^#^ vs. control group (delta changes); *P* < 0.05.

The effects of IMT and AET on LBF and LVC, respiratory muscle strength, functional capacity, and quality of life are shown in *Figure* 5. Both interventions increased LBF (IMT, *P* = 0.04, and AET, *P* = 0.02, *Figure* 5A). In regard to LVC, the effect of AET on LVC was more pronounced than those found in IMT (*P* = 0.03 and *P* = 0.07, respectively, *Figure* 5B). Further comparisons showed that the changes in LBF caused by AET and IMT were greater than those found in the control group (*P* = 0.01 and *P* = 0.05, respectively, *Figure* 5A). Similarly, the changes in LVC provoked by AET and IMT were greater than those in the control group (*P* = 0.05 and *P* = 0.02, respectively, *Figure* 5B).

As expected, IMT increased maximal inspiratory pressure (*P* = 0.0002, *Figure* 5C). No significant changes in maximal inspiratory pressure were observed in the control and AET groups.

In relation to functional capacity, both AET and IMT significantly increased peak VO_2_ (*P* = 0.001 and *P* = 0.01, respectively, *Figure* 5D). No changes were found in the control group. Thus, the percent change analysis showed that the changes in peak VO_2_ provoked by AET (*P* = 0.001) and IMT (*P* = 0.02) were greater compared with the control group (*Figure* 5D). The peak workload significantly increased in the three groups studied (control, *P* = 0.03; IMT, *P* = 0.01; and AET, *P* = 0.002, respectively, *Figure* 5E). However, the changes in peak workload in AET were greater than those found in IMT and control groups (*P* = 0.004, *Figure* 5E). Further analyses showed a significant correlation between percent changes in follistatin mRNA and percent changes in peak VO_2_ (*P* = 0.004, rho = 0.51).

Both IMT (*P* = 0.001) and AET (*P* = 0.001) significantly decreased MLHFQ score, which means an improvement in quality of life (*Figure* 5F). In contrast, the MLHFQ score significantly increased in the control group (*P* = 0.01, *Figure* 5F). The percent change comparisons among groups showed that the changes caused by IMT and AET in quality of life (*P* = 0.0001 and *P* = 0.0001, respectively, *Figure* 5F) were significantly different from those found in the control group.

## Discussion

**Figure 6:**
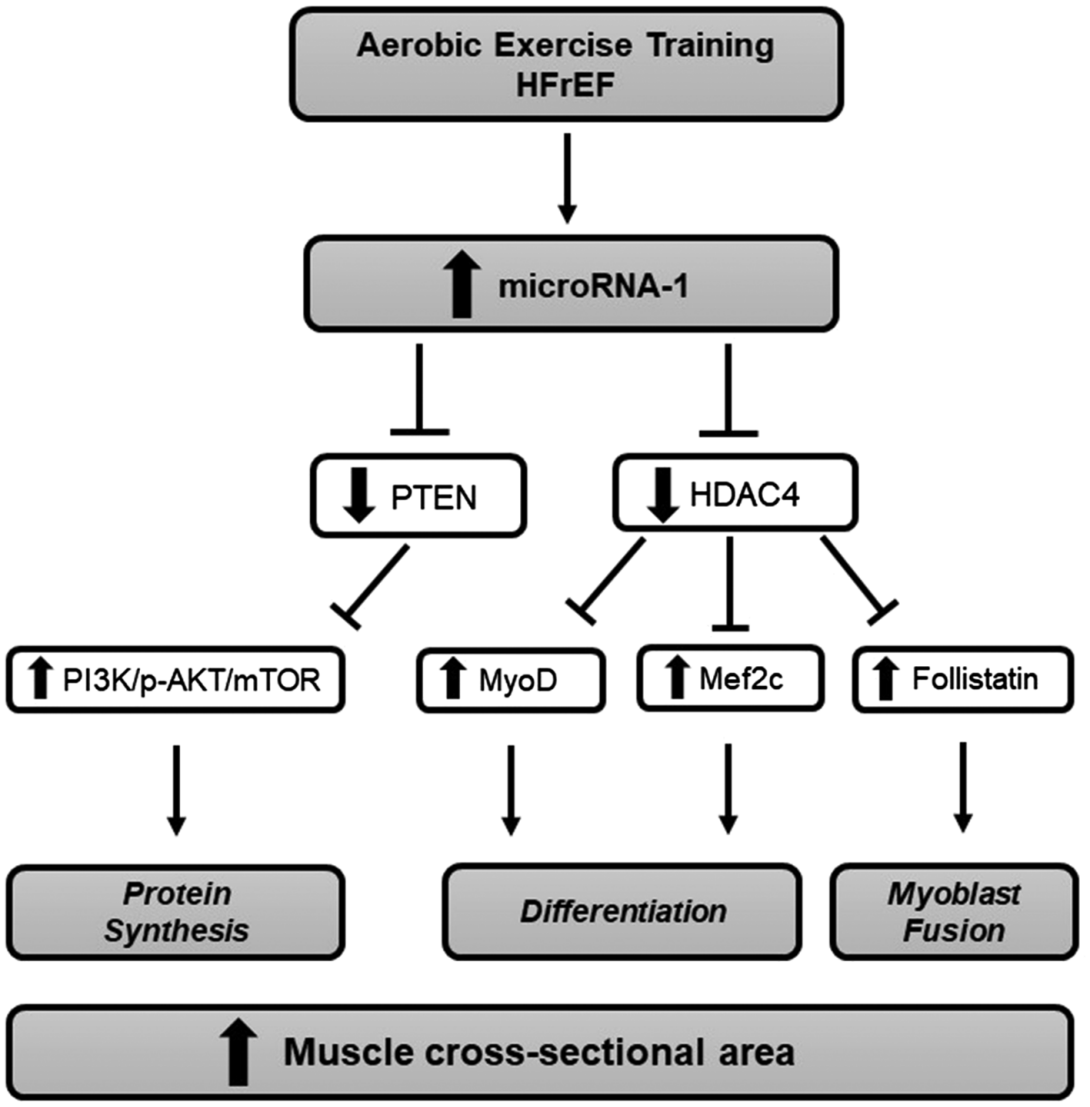
Changes provoked by aerobic exercise training on microRNA‐1 downstream‐associated pathways in patients with chronic heart failure with reduced ejection fraction (HFrEF). Note that despite the increase in microRNA‐1 levels, the PI3K‐AKT pathway is increased, which seems to be associated with a reduction in PTEN levels. In addition, HDAC4 levels decrease, which leads to an increase in follistatin, MEF2C, and MyoD levels. These changes improve protein synthesis, skeletal muscle differentiation, and myocyte fusion. The consequence of such changes is an increase in muscle cross‐sectional area.

The main and new findings of the present study are that AET, but not IMT, improves microRNA‐1 expression and downstream‐associated pathways in patients with chronic HFrEF. AET increases microRNA‐1 levels and decreases the expression of PTEN, which in turn reduces its inhibitory action on PI3K‐AKT pathway. In addition, AET decreases HDAC4 levels, which results in an increase in the levels of follistatin, MEF2C, and MyoD. These molecular responses, associated with the increase in muscle fibre cross‐sectional area (*Figure* 6), certainty contribute to the amelioration in the skeletal myopathy in chronic heart failure.

Some investigators reported that AET reduces microRNA‐1 levels and increases PI3K/AKT/mTOR pathways in healthy people.^47^ A different scenario was observed in patients with skeletal myopathy associated with chronic HFrEF. AET increased the levels of PI3K and p‐AKT, despite the augmentation in the expression of microRNA‐1. These findings corroborate the idea that the PI3K/AKT/mTOR pathway was activated post‐exercise training. Based on this observation, we raised the hypothesis that the increased levels of p‐AKT would be associated with a reduction in PTEN expression. Although PTEN is not a predicted target gene of microRNA‐1, there is evidence that increased microRNA‐1 levels downregulate PTEN levels.^48^, ^49^ The reduction in PTEN in our study favoured the increase in p‐AKT stimulating protein synthesis by mTOR upregulation. Moreover, these responses seem to contribute to an improvement in anabolic/catabolic balance expressed by the enhancement in the muscle fibre cross‐sectional area.^3^, ^5^, ^26^, ^27^, ^50^ At this point, the explanation for the differences in the microRNA‐1 expression provoked by exercise training in healthy individuals and patients with chronic HFrEF remains uncertain. Surely, this topic deserves future investigation.

Increased levels of microRNA‐1 can be also involved in postnatal myoblast differentiation by suppressing HDAC4, a predicted target gene of this myomiR.^7^, ^10^, ^11^, ^12^, ^51^, ^52^, ^53^ HDACs are well known for regulating muscle proliferation, differentiation, and growth through the regulation of histone acetylation. In particular, class IIa HDACs (HDACs 4, 5, 7, and 9) plays an important role in the maintenance of muscle mass and protein degradation during muscle wasting.^54^ The reduced levels of HDAC4 promote myoblast differentiation, while increased levels of HDAC4 enhances skeletal muscle atrophy.^7^, ^10^, ^12^, ^51^, ^55^, ^56^, ^57^, ^58^ Chen *et al*.^12^ reported that overexpression of microRNA‐1 strongly repressed HDAC4 expression, which results in myoblast differentiation in myotube via HDAC4/MEF2/MyoD family pathways in C2C12 cells. In the present study, we found that AET downregulated HDAC4 expression likely associated with the increased levels of microRNA‐1. These molecular responses led to an increase in MEF2c and MyoD mRNA levels, which results in stimulation of skeletal muscle satellite cells differentiation. These responses highlight the importance of AET in counteracting the skeletal muscle loss in chronic HFrEF.

Another important finding in the present study is the increased follistatin mRNA levels after AET. Previous studies have demonstrated a significant relationship between follistatin and muscle growth and/or hypertrophy.^59^, ^60^ Actually, Sun *et al*.^11^ showed that increased levels of *mammalian target of rapamycin* (mTOR) upregulate MyoD expression and microRNA‐1 transcription, which suppresses HDAC4 and subsequently increases follistatin. This myogenic pathway mTOR/MyoD/microRNA‐1/HDAC4/follistatin stimulates skeletal myoblast fusion. Our study shows that AET increases the follistatin expression by increasing microRNA‐1 levels and by upregulating mTOR/MyoD feedback on microRNA‐1, which is important stimuli to myoblast fusion. In fact, the increase in microRNA‐1 levels was associated with the increase in follistatin levels (*P* = 0.001, rho = 0.58). In addition, there was a tendency toward association between the increase in MyoD levels and the increase in follistatin levels (*P* = 0.06, rho = 0.35). More importantly, these findings have the potential for clinical application. Reduced levels of follistatin are associated with atrophy and skeletal myopathy in animal model of HF.^61^ The increase in follistatin levels contributes to the reduction in myostatin levels, a negative regulator of muscle mass. Some authors previously showed that AET reduces myostatin levels and contributes to the increase in muscle mass and exercise capacity in animal model of HF.^50^ Our study shows a correlation between the increase in follistatin levels and the improvement in peak VO_2_ in patients with HFrEF.

Exercise intolerance in patients with chronic HFrEF has been attributed, in great part, to skeletal myopathy. Exercise training has been shown to counteract the muscular alterations in this syndrome by improving many of the key features of the skeletal myopathy.^3^, ^5^, ^26^, ^27^ This nonpharmacological strategy increases muscle capillarization, muscle blood flow, and flow‐mediated dilation, which facilitate oxygen diffusion and oxidative energy production.^3^, ^5^, ^26^, ^27^ In addition, exercise training ameliorates protein degradation pathways by downregulating the ubiquitin‐proteasome system and stimulating insulin‐like growth factor‐1 (IGF‐1) signalling pathway.^3^, ^5^, ^26^, ^27^ These changes contribute to the restoration in anabolic/catabolic imbalance.^3^, ^5^, ^26^, ^27^ The present study extends the knowledge that AET improves the microRNA‐1 downstream‐associated pathways in patients with chronic HFrEF, which seems to improve skeletal myopathy and exercise capacity in this set of patients.

Accumulated evidence shows that exercise training increases muscle blood flow in chronic heart failure.^16^, ^17^, ^18^, ^19^, ^20^, ^21^, ^22^, ^23^, ^24^, ^25^ Our study confirms this finding. AET increased LBF in patients with chronic HFrEF. This observation has clinical implications because muscle blood flow is an independent predictor of mortality in these patients.^62^ However, our study provides no information regarding the mechanisms involved in the increase of LBF. We speculate that increase in endothelial function and reduction in sympathetic nerve activity contribute to the amelioration in LBF. Previous studies demonstrate that AET increases endothelial‐mediated blood flow and reduces muscle sympathetic nerve activity in chronic heart failure.^4^, ^5^, ^16^, ^17^, ^18^, ^19^, ^20^, ^21^, ^22^, ^23^, ^24^, ^63^, ^64^ IMT also increased LBF,^33^, ^65^ but the mechanisms underlying this response are virtually unknown. This issue is an interesting topic for future investigations.

Increase in functional capacity after exercise training has been reported in patients with chronic HFrEF regardless of age, gender, and aetiology.^16^, ^17^, ^19^ Our study is consistent with this observation. AET increased peak VO_2_ and peak workload. We have no explanation for this response. However, it is conceivable that the increase in LBF and the improvement in skeletal myopathy contributed to the enhancement in the functional capacity in our patients.

Premature inspiratory muscle fatigue stimulates pulmonary ventilation.^66^ This response activates inspiratory muscle metaboreceptors that reflexively increase sympathetic nerve activity.^66^ The augmentation in sympathetic outflow causes skeletal muscle vasoconstriction that contributes to early fatigue.^66^ Our study shows that IMT increases peak VO_2_ in patients with chronic HFrEF. It is possible that IMT by enhancing global inspiratory muscle strength, represented by maximal inspiratory pressure, delays diaphragm fatigue, attenuating the reflex sympathetic nerve activity discharge and skeletal muscle vasoconstriction. In fact, we found that IMT increases LBF, and reduction in muscle sympathetic nerve activity was reported after IMT in patients with HF.^37^ More recently, Smuder *et al*. showed that exercise increases the expression of heat shock protein 72 (HSP72), which reduces the expression of atrophic transcript factors and oxidative stress. These responses preserve diaphragm muscle function in animals with diaphragm dysfunction.^67^ Molecular adaptations in respiratory muscles were out of the scope of our study. However, it is possible that the increase in HSP72 levels is involved in inspiratory muscle adaptation in patients with chronic HFrEF. In summary, IMT improves inspiratory muscle strength and neurovascular control, increases skeletal muscle perfusion, and provokes respiratory muscle adaptations. The consequence of such changes is an increase in exercise tolerance in patients with HFrEF.^33^, ^66^, ^68^, ^69^ These findings support the concept of IMT as a strategy in the treatment for patients with HFrEF.

AET caused a remarkable change in microRNA‐1 expression and downstream‐associated pathways in leg muscle. These changes were not observed in the patients enrolled in the IMT. In addition, the increase in LVC and the increase in muscle fibre cross‐sectional area were more pronounced in patients involved in AET than in patients involved in IMT. These findings can be attributed to the specificity of local training effects on exercise muscle. AET was performed on a bicycle ergometer, whereas IMT consisted of breathing against resistance.

Despite its recognized benefits, exercise‐based cardiac rehabilitation has been an underused therapy.^70^ There are many potential barriers, including socio‐economic factors, work conflicts, inadequate transportation, and patient attitude and motivation, that affect the adherence of patients with HFrEF to cardiac rehabilitation programmes. In a recent report, the European Society of Cardiology provides practical recommendations on how to implement exercise training for heart failure patients with ventricular assistance devices.^71^ The benefits of exercise training in these patients are not fully understood, but it seems to be a promising therapy for clinical improvement. Exercise training increases functional capacity and decreases N‐terminal pro‐B‐type natriuretic peptide (NT‐proBNP) in heart failure patients.^71^ Alternative therapies based on IMT may be useful in cardiac rehabilitation programmes if the patient is unable to perform conventional exercise training. IMT provokes remarkable benefits for patients with HFrEF. In addition, recent reports show that electro‐myostimulation is an important strategy to improve muscle structure, function, and atrophy in patients with chronic HErEF.^72^

We recognize several limitations in our study. Respiratory muscle biopsy would improve the understanding regarding the effects of IMT and AET on functional capacity. The small sample size may limit our interpretation, but this is a very complex and herculean study that is unlikely to be repeated. Fourty‐four HFrEF patients were randomized into control group, IMT group, and AET group. Three patients in the IMT and two patients in the AET were excluded because of HF decompensation or cardiovascular events. Although these exclusions may generate bias in the study, patients that were hospitalized were not able to proceed with the training protocol due to patient safety issues. The responses achieved by the association of both IMT and AET are not available. It is possible that benefits of both IMT and AET on skeletal myopathy, muscle blood flow, and functional capacity are even greater than AET and IMT alone.

In conclusion, AET upregulates microRNA‐1 levels. The increased levels of microRNA‐1 decrease the protein expression of PTEN, which in turn reduces the inhibitory action on the PI3K‐AKT pathway that regulates the skeletal muscle tropism. In addition, increased levels of microRNA‐1 decrease HDAC4 and upregulate MEF2c, MyoD, and follistatin levels, improving skeletal muscle regeneration. These changes in skeletal muscle phenotype associated with the increase in the muscle cross‐sectional area and LBF contribute to the attenuation in skeletal myopathy and improves the functional capacity in patients with HFrEF. IMT also increases functional capacity. However, this response seems to be associated with amelioration in the respiratory function instead of changes in skeletal muscle.

## Conflict of interest

The author(s) declare(s) that there is no conflict of interest.

## Funding

This study was supported by the Fundação de Amparo à Pesquisa do Estado de São Paulo (FAPESP#2015/22814‐5) and, in part, by Fundação Zerbini. Ligia M. Antunes‐Correa was supported by FAPESP (#2013/15651‐7). Patricia C. Brum, Edilamar M. de Oliveira, and Carlos Eduardo Negrão were supported by CNPQ (#306261/2016‐2, #313479/2017‐8, and #303573/2015‐5).
